# Determination of the Mechanisms of Terbium(III) Biosorption by *Bacillus* Strains with Adsorption Selectivity for Heavy Rare Earth Elements

**DOI:** 10.3390/microorganisms13081753

**Published:** 2025-07-27

**Authors:** Huihong Huang, Kang Pan, Wenchao Jian, Yuwen She, Comfort O. Esumeh, Wei Dong

**Affiliations:** 1 Jiangxi Provincial Key Laboratory of Environmental Pollution Prevention and Control in Mining and Metallurgy, Ganzhou 341000, Chinacomfortesumeh@gmail.com (C.O.E.); 2 School of Resources and Environmental Engineering, Jiangxi University of Science and Technology, Ganzhou 341000, China; 3 School of Life Sciences, Jiangxi University of Science and Technology, Ganzhou 341000, China; 4 Yichun Lithium New Energy Industry Research Institute, Jiangxi University of Science and Technology, Yichun 336000, China

**Keywords:** biosorption, Tb(III), heavy rare earth elements, *Bacillus*, functional groups

## Abstract

*Bacillus* species have shown the potential to recover rare earth elements (REEs), but strains with adsorption selectivity for terbium(III) remain understudied. In this study, six *Bacillus* strains with the capability for efficient adsorption of Tb(III) were screened from an ionic rare earth mine and were identified based on 16S rRNA gene sequencing. Adsorption experiments showed that *Bacillus* sp. DW011 exhibited exceptional Tb(III) adsorption efficiency, with an adsorption rate of 90.45% and adsorption selectivity for heavy rare earth elements. Notably, strain DW011 was also found to be tolerant against Tb(III) with the 24 h 50% lethal concentration (LC_50_) of 2.62 mM. The biosorption mechanisms of DW011 were investigated using adsorption kinetics, SEM-EDS, and FTIR. The results indicated that the adsorption of strain DW011 conforms to the second-order kinetic model, and the teichoic acid–peptidoglycan network (phosphate-dominated) serves as the primary site for heavy REE adsorption, while carboxyl/amino groups in the biomembrane matrix provide secondary sites for LREEs. This study provides new information that *Bacillus* strains isolated from ionic rare earth mine deposits have potential as green adsorbents and have high selectivity for the adsorption of heavy REEs, providing a sustainable strategy for REE recovery from wastewaters.

## 1. Introduction

Rare earth elements (REEs) encompass scandium, yttrium, and 15 lanthanide elements. Due to their distinctive physicochemical properties, these elements are extensively utilized in the research and development of high-tech products, including batteries, magnetic, materials, smartphones, and aircraft [1,2,3,4]. Recently, the demand for REEs has grown exponentially due to the rapid development of high-tech industries that rely heavily on rare earths [5]. However, with intensive mining and extensive application, REEs have been released into the environment, particularly in water bodies, potentially leading to ecological pollution [6,7]. REEs can also be transmitted and accumulated along the food chain, causing harm to microorganisms, plants, animals, and humans [8,9,10,11,12]. Consequently, recovering rare earths from wastewater can alleviate the impact of rare earth pollution on aquatic ecosystems and human health, and hence meet the growing global demand for these elements. Researchers have employed numerous techniques to treat rare earth-containing water bodies, including precipitation, ion exchange, electrochemistry, reverse osmosis, and adsorption resins [13,14,15,16,17]. However, these conventional methods face limitations such as high costs, the risks of secondary pollution, and low recycling efficiency, which restrict their applicability [18,19,20]. Therefore, it is urgent to develop green and efficient methods to recover REEs. Microbial adsorption is regarded as a sustainable and efficacious approach for REE recovery [19,21,22,23].

Compared with traditional chemical methods, microbial adsorption exhibits several advantages, including cost-effectiveness, enhanced environmental compatibility, and the possibility of reusable adsorbents [24,25,26]. A number of studies have demonstrated that algae, fungi, and bacteria have remarkable REE adsorption capabilities [27,28,29]. Paper et al. reported that cyanobacteria exhibited saturated adsorption capacities ranging from 84.2 to 91.5 mg/g for cerium, neodymium, terbium, and lanthanum at a concentration of 10 mM [30]. The fungus ZD28 exhibited excellent adsorption capacity for Y(III), exceeding 455 μmol/g, within a concentration range from 465 μM to 6382 μM [31]. *Escherichia coli* demonstrated an adsorption capacity of 41.9 mg/g for Tb(III) at a concentration of 150 μM [32]. Similarly, the adsorption of REEs by *Bacillus* has been demonstrated to exhibit considerable ability. Cheng et al. discovered that *Bacillus licheniformis* could achieve a saturation adsorption capacity of 38.93 mg/g for cerium in an initial solution with a concentration of 200 mg/L [33]. Moriwak et al. reported that *Bacillus subtilis* 168 powder exhibited a high adsorption capacity of 43.1 mg/g for thulium [34]. Tsuruta et al. confirmed that *Bacillus* strains exhibited a higher adsorption capacity for rare earth ions than other strains [35]. Moreover, the spores produced by *Bacillus* maintain effective biosorption performance for rare earth ions even in extreme environments [36]. Despite these advances, the number of currently applied strains for the recovery of rare earths is still relatively limited, highlighting the necessity for continued screening of strains with efficient adsorption capacity for REEs [37].

Terbium, as a critical rare earth element, plays an indispensable role in biomedical equipment, fluorescent lamps, and batteries [3,38]. Particularly in high-performance magnetic materials, the incorporation of terbium significantly enhances the coercivity and thermal stability of NdFeB permanent magnets, making it essential for electric vehicle traction motors and wind turbine generators [2]. With the rapid expansion of the global EV industry and sustained growth in wind power installations, the demand for Tb in permanent magnet applications is experiencing exponential growth [39]. However, Tb is one of the scarcest rare earth metals, and over-exploitation has led to multiple limitations on its supply [23]. Thus, developing efficient Tb recovery technologies holds significant importance for ensuring resource security and advancing green low-carbon industries.

In this study, *Bacillus* strains were isolated and screened to obtain biosorbents with a high adsorption capacity for REEs, especially for Tb. Strain selection was conducted using soil samples from rare earth mining sites in southern Jiangxi, China. Subsequently, the *Bacillus* strains with the highest adsorption capacity for Tb(III) were selected and identified by systematic phylogenetic analysis, and their adsorption selectivity for different REEs was determined. Detailed studies were conducted of the strains with the most optimal adsorption selectivity, including evaluation of their tolerance to Tb(III) and adsorption mechanisms. The principal mechanism underlying the rare earth adsorption process was elucidated through a combination of adsorption kinetics, scanning electron microscopy with energy-dispersive X-ray spectroscopy (SEM-EDS), and Fourier transform infrared spectroscopy (FTIR) characterization analysis.

## 2. Materials and Methods

### 2.1. Screening and Isolation of Bacillus Strains

Soil samples were collected from a rare earth mine in southern Jiangxi, China. A 10 g mass of each soil sample was added to a triangular bottle containing glass beads and 90 mL of sterile water. Then, 1 mL of supernatant from the soil solution was transferred to a container. Subsequently, the solution was heated in a water bath at 90 °C for 10 min and cooled in an ice bath for 15 min. Subsequently, 100 μL was diluted in a gradient (10^−1^ to 10^−5^) and spread on LB solid medium (1% peptone, 1% NaCl, 0.5% yeast extract, and 1.8% agar) containing 6.3 mM Tb(III). The colonies were then incubated overnight at 37 °C to obtain single colonies for the primary screening process. Single colonies were selected and streaked three times on LB solid medium to isolate the microorganisms and obtain pure cultures.

### 2.2. Culture of Bacillus Strains

A bacterial suspension was prepared from the activated microorganisms. A 1 mL volume of the bacterial suspension was added into 150 mL of LB medium (1% peptone, 1% NaCl, and 0.5% yeast extract) and cultured at 37 °C and 200 rpm for 8 h. The concentration of the bacterial solution was determined by measuring its absorbance at a wavelength of 600 nm (OD_600_) using a UV spectrophotometer (755B, Shanghai Youke Instrument Co., Ltd., Shanghai, China).

Following centrifugation of the suspended cells, the precipitates obtained were subjected to cryogenic cooling at −80 °C for 6–8 h in an ultra-low-temperature refrigerator, and then put into a SCIENTZ-10N vacuum freeze-dryer (Ningbo Scientz Biotechnology Co., Ltd., Ningbo, China) for 24 h to dry them into a powder.

### 2.3. Screening of Bacillus Strains with Efficient Adsorption for Tb(III)

The adsorption capacity of *Bacillus subtilis* PS832, a model strain from UConn Health belonging to the American Academy of Microbiology fellow Peter Setlow, was compared with that of 24 wild microorganisms isolated from mine soil. It was selected as a comparison microorganism not only for its genetic stability, but also because it shows efficient adsorption of Tb ions [40]. The experimental procedure was carried out as described, except that we used bacterial liquid instead of powder [40]. Specifically, 125 µL of 100 mM 2-[4-(2hydroxyethyl)-1-piperazinyl] ethane sulfonic acid (HEPES) at pH 7.4, 50 µL of 2 mM Tb(III) solution, 75 µL of sterile water, and 250 µL of bacterial solution with an OD_600_ of 3.0 were mixed and allowed to stand for 5 min, and then they were centrifuged at 12,000 rpm for 2 min. Three replicates were set up for each group. The fluorescence intensity of the rare earth ions in the solution was measured before and after adsorption by *Bacillus*, and the concentration was calculated according to the Tb(III) concentration standard curve. The adsorption rate *R* (%) was calculated according to Equation (1):

(1) $$R=\left(1-\frac{C}{C_{0}}\right)\times 100\%$$

where *C*_0_ is the concentration of rare earth ions in the solution before adsorption (μM), and *C* is the concentration of rare earth ions in the solution after adsorption (μM).

### 2.4. Phylogenetic Characterization of Bacillus Strains

Following the rare earth adsorption screening experiments, strains with robust Tb(III) adsorption capacity were selected for activation. Genomic DNA was extracted from these activated cultures using the FastDNA SPIN Kit (MP Biomedicals, Irvine, CA, USA). The 16S rRNA gene was amplified from the extracted DNA using the universal primers 27F (5′-AGAGTTTGATCCTGGCTCAG-3′) and 1492R (5′-GGTTACCTTGTTACGACTT-3′). Amplified products were sequenced by Sangon Biotech (Shanghai, China). The resulting sequences were compared to the NCBI database via BLAST online tool, and phylogenetic trees were constructed by MEGA 11 using the Neighbor-Joining algorithm with the *p*-distance model (bootstrap = 1000 replicates).

### 2.5. Screening of Bacillus Strains with Selective Adsorption

The adsorption performance of bacterial suspensions at OD_600_ = 1.5 was comparable to that of 2 g/L bacterial powder based on preliminary studies. However, the adsorption capacity of the bacterial suspensions significantly deteriorated due to their poor stability after just 24 h of storage. To ensure the reproducibility and replicability of the results, we therefore employed bacterial powder for all subsequent selective adsorption experiments with *Bacillus* strains. A mixture containing 2.5 mL of 100 mM HEPES at pH 7.4, 7.5 mg of bacterial powder, 1 mL of rare earth mixed solution, and 6.5 mL of sterile water was allowed to stand for 10 min. The rare earth mixed solution contained 1 mM each of Tm(III), Ho(III), Y(III), Dy(III), Nd(III), Ce(III), and La(III). Subsequently, the supernatant was subjected to centrifugation and filtration through a filter membrane with a pore size of 0.22 μm. Following this, Inductively Coupled Plasma Mass Spectrometry (ICP-MS; Agilent Technologies China Co., Ltd., Beijing, China) analysis was conducted in accordance with the specified analytical methods, and the adsorption rate *R* (%) was calculated in accordance with the specified Equation (1).

### 2.6. Analytical Methods

#### 2.6.1. Measurement of Tb(III)

Rare earth ions form fluorescent complexes with 2,6-pyridinedicarboxylic acid (DPA), exhibiting fluorescence intensities that correlate linearly with the concentration of rare earth ions within a certain range [40]. The reaction mixture consisted of 90 µL of the solution to be tested, 100 µL of 100 mM HEPES buffer, and 10 µL of 10 mM DPA solution. The fluorescence intensity was quantified at an excitation wavelength of 275 nm and an emission wavelength of 545 nm. Subsequently, the fluorescence intensities obtained were substituted into the standard curve to determine the concentration of rare earths in the solution under investigation. The experiment was conducted using a multifunctional enzyme marker for determination.

#### 2.6.2. Measurement of Mixed Rare Earth

The concentration of REEs in the solution before and after adsorption was determined using ICP-MS. This resulted in the determination of mixed rare earth ion concentrations.

#### 2.6.3. Statistical Analysis

All experiments were performed in triplicate (*n* = 3) with three independent biological replicates. Data were expressed as the mean ± standard deviation (SD). Statistical significance was determined using one-way analysis of variance (ANOVA) followed by Tukey’s honestly significant difference (HSD) post hoc test for multiple comparisons. The threshold for statistical significance was 0.05. All statistical analyses were performed using GraphPad Prism 9.0 (GraphPad Software, San Diego, CA, USA).

### 2.7. Characterization of DW011

#### 2.7.1. Test of Tolerance Against Tb(III)

The bacterial seed solution, which was cultured at 180 rpm and 37 °C for 12 h, was inoculated into LB liquid medium containing 0, 1.5, 2.3, 2.6, 3.0, and 3.8 mM TbCl_3_, with an inoculation volume of 2.5% (*v*/*v*). Subsequently, the cultures were placed in a constant-temperature shaker at 200 rpm and 37 °C for cultivation. The optical density of the culture broth was measured at OD_600_ every hour until a stable reading was obtained.

#### 2.7.2. Adsorption Kinetic Models

The adsorption kinetics experiment was conducted by varying the adsorption time of the bacterial solution (OD_600_ = 3.0) in Tb(III) solution. The adsorption system consisted of 250 μL of the bacterial solution, 50 μL of the 2 mM Tb(III) solution, 125 μL of the 100 mM HEPES, and 75 μL of sterile water. The experiment was conducted at a controlled temperature of 25 °C, with adsorption times set at 1, 3, 5, 10, 15, and 20 min. Following centrifugation, the supernatant was analyzed to determine the concentration of Tb(III) using the method described in Section 2.6.1. The pseudo-first-order model and pseudo-second-order model were employed to fit the experimental results, the linear expressions of which are given by Equations (2) and (3), respectively:

(2) $$\begin{array}{c} ln\left(q_{e}-q_{t}\right)={ln\;q}_{e}-K_{1}t \end{array}$$

(3) $$t/q_{t}=1/K_{2}q_{e}^{2}+t/q_{e}$$

where *q_e_* and *q_t_* (mg/mL) are the equilibrium adsorption capacity and the dynamic adsorption capacity (at time *t*), *K*_1_ is the pseudo-first-order kinetic adsorption rate constant, and *K*_2_ is the pseudo-second-order kinetic adsorption rate constant.

#### 2.7.3. SEM-EDS Analysis

The post-adsorption solution was centrifuged (8000 rpm, 10 min), the supernatant was discarded, and the precipitate was collected. The bacterial precipitate was washed three times with deionized water, pre-cooled at −20 °C and −80 °C respectively, and then dried in a vacuum freeze-dryer for 24 h. The lyophilized powder was uniformly dispersed onto conductive tape and coated with gold using an ion sputter coater. After coating with gold, the sample surface morphology was analyzed using an SEM (MLA650, FEI, Hillsboro, OR, USA) and EDS (Esprit 1.9, Bruker, Berlin, Germany) at an acceleration voltage of 20 kV.

#### 2.7.4. FTIR Analysis

Before and after adsorption, precipitates were washed three times with sterile water and then dried using a vacuum freeze-dryer, before being mixed with KBr at a sample/KBr weight ratio of 1:100. A thin pellet was placed in the IR beam on an FT-IR spectrometer (Nicolet iS5, Thermo Scientific, Madison, WI, USA) and scanned over a range of 4000–400 cm^−1^ with a step size of 4 cm^−1^.

## 3. Results and Discussion

### 3.1. Bacillus Strains with Efficient Adsorption for Tb(III)

**Figure 1:**
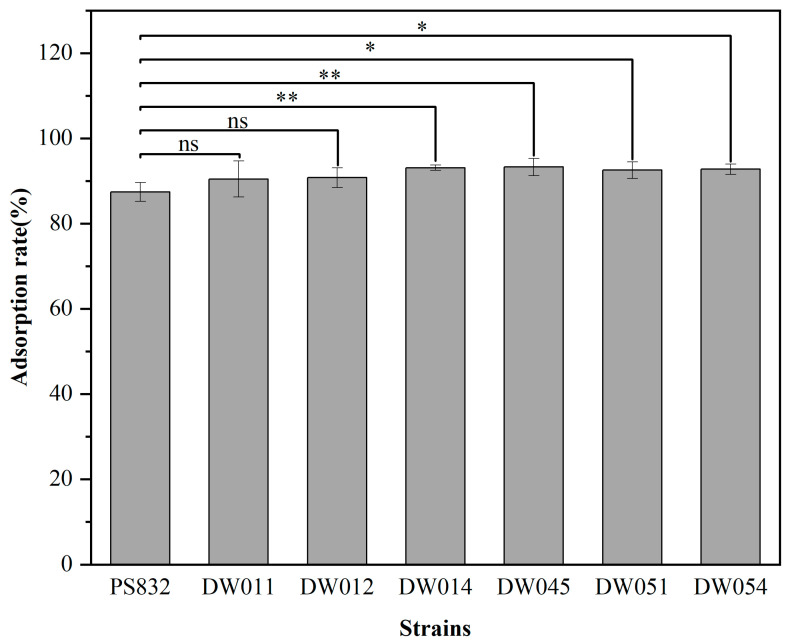
The adsorption rate of different strains with OD_600_ of 1.5 in 200 μM Tb(III) for 5 min at pH 7.4. The adsorption rate for each strain was analyzed in three independent experiments in triplicate, and the data are shown as the mean ± SD. *, **, and ns indicate significant differences at the 0.05 level, significant differences at the 0.01 level, and no significant differences, respectively.

*B. subtilis* PS832 and the 24 bacterial strains isolated from rare earth ore soil were incubated with Tb(III) at a concentration of 200 μM at pH 7.4. The results showed that the adsorption rate after 5 min for six strains was comparable to that of *B. subtilis* PS832 (Figure 1). Specifically, strains DW011, DW012, DW014, DW045, DW051, and DW054 exhibited Tb(III) adsorption rates of 90.45%, 90.77%, 93.10%, 93.28%, 92.53%, and 92.74%, respectively. The comparison analysis showed that the Tb(III) adsorption efficiency of four strains (DW014, DW045, DW051, DW054) was significantly higher than that of the reference strain PS832, while there was no significant difference between the other two strains (DW011 and DW012) and the reference strain.

A number of studies have demonstrated that microorganisms can adsorb rare earth ions onto cell walls and membranes through reactive groups on the cell surface [41]. However, adsorption capacities vary substantially across strains due to the factors influenced by strain genetic background, cell wall structure, metabolic activity, and extracellular polysaccharide production [42]. Compared to the wild-type *Bacillus subtilis*, the lipoteichoic acid-defective strains exhibited significantly reduced adsorption of La(III), Eu(III), and Tm(III) [34]. Therefore, variations in functional groups on the cell surfaces of these strains may explain the differences in adsorption capacity observed in this study. Furthermore, environmental stress can facilitate the emergence of bacterial resistance mutations [43]. Under high-salinity conditions, bacteria produce more biofilms to enhance salt resistance, leading to a greater diversity of functional groups on their cell surfaces [44]. This mechanism may explain why efficient rare earth-adsorbing strains were obtained from rare earth mine soils in this study. In subsequent experiments, these six bacterial strains which exhibited the highest adsorption rates were selected for further screening.

### 3.2. Phylogenetic Characterization of Bacillus Strains

**Figure 2:**
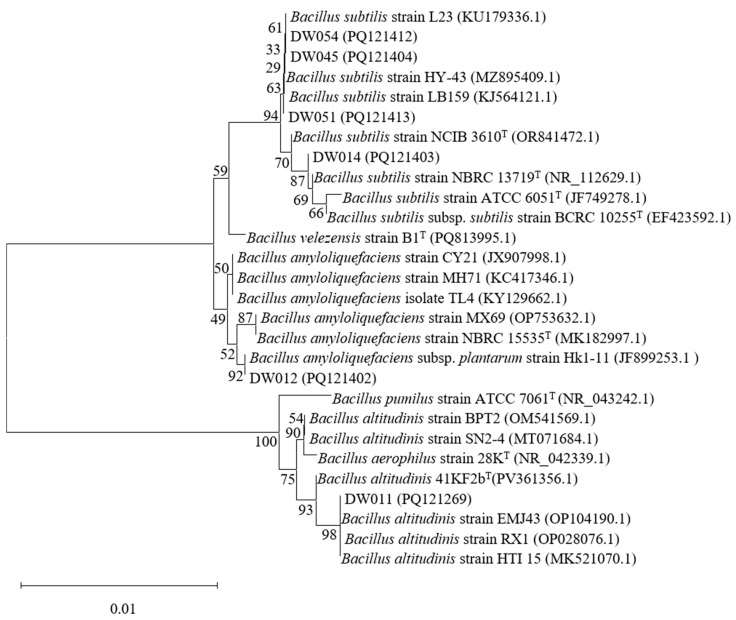
Phylogenetic analysis of screened *Bacillus* strains based on 16S rRNA gene sequencing. T indicates the type strain.

Phylogenetic tree analyses were conducted for six high-efficiency rare earth-adsorbing strains to identify *Bacillus* species among them. The 16S rRNA sequences of strains DW011, DW012, DW014, DW045, DW051, and DW054 were analyzed using the NCBI BLAST online tool (https://blast.ncbi.nlm.nih.gov/, accessed on 4 July 2025) to determine their phylogenetic positions (Figure 2). Significantly, due to the high conservation of 16S rRNA sequences in *Bacillus*, these strains were identified as *Bacillus* spp. based on similarity percentages between the studied strains and their closest type strains. First and foremost, DW011 showed 100% similarity to *Bacillus altitudinis* 41KF2b^T^ (PV361356.1), 99.86% similarity to *B. aerophilus* 28K^T^ (NR_042339.1), and 99.51% similarity to *B. pumilus* ATCC 7061^T^ (NR_043242.1). Phylogenetic analysis classified DW011 within the *B. altitudinis* clade, though the high sequence similarity among species in the *B. pumilus* group warranted further genomic confirmation. Similarly, DW012 exhibited 99.66% similarity to *B. amyloliquefaciens* NBRC 15535^T^ (MK182997.1) and 99.45% similarity to *B. velezensis* B1^T^ (PQ813995.1), suggesting its affiliation with the *B. amyloliquefaciens* group. Furthermore, DW014, DW045, DW051, and DW054 shared 99.45–99.93% similarity with *B. subtilis* type strains (e.g., *B. subtilis* NCIB 3610^T^, ATCC 6051^T^) and 99.37–99.52% similarity with *B. velezensis* B1^T^, indicating their close relationships with the *B. subtilis* species complex. In conclusion, all six strains were confidently assigned to the *Bacillus* genus, but their exact species classification requires further identification.

### 3.3. Bacillus Strains with Selective Adsorption

**Figure 3:**
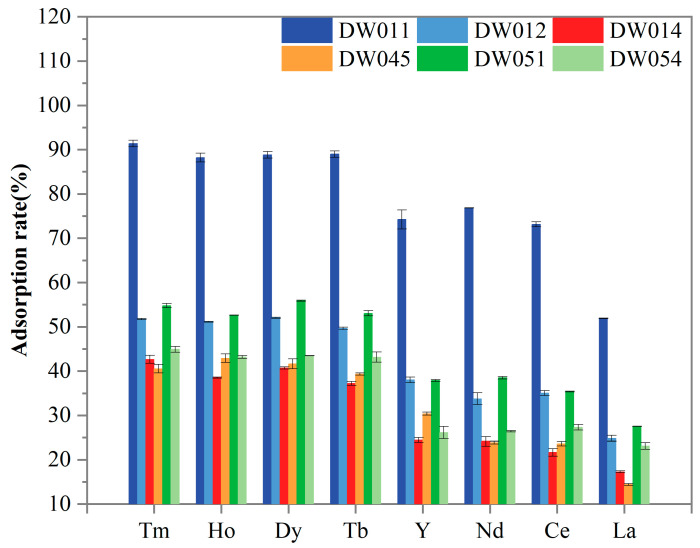
Comparison of adsorption rate of different *Bacillus* powders for REEs. Adsorption rate for each strain was analyzed in three independent experiments in triplicate, and data are shown as mean ± SD.

Further screening was conducted to evaluate the adsorption selectivity of six *Bacillus* strains towards Tm(III), Ho(III), Y(III), Dy(III), Tb(III), Nd(III), Ce(III), and La(III). As presented in Figure 3, all the tested REEs were adsorbed by the strains. The statistical analysis revealed striking differences in REE adsorption capabilities among the six bacterial strains (Table 1). Strain DW011 consistently demonstrated the highest adsorption efficiency (group a), significantly outperforming other strains. Strains DW012 and DW051 generally showed moderate adsorption rates, forming an intermediate group b, while DW014, DW045, and DW054 typically exhibited the lowest adsorption capacities (group c/d). Furthermore, paired t-tests with FDR correction revealed significant preferential adsorption of certain heavy rare earth elements (HREEs: Tm, Ho, Dy, Tb, and Y in this study) over light rare earth elements (LREEs: Nd, Ce, and La in this study) across all strains (Table 1). Specifically, Tm, Ho, Dy, and Tb consistently formed the higher-adsorption group for each strain (α-γ), while LREEs occupied significantly lower-adsorption groups (γ-ζ). DW011 exhibited the most pronounced differentiation, with Tm-Ho-Dy-Tb at 88–91% (α-β) ver-sus 51–76% for LREEs (γ-δ). Notably, although Y is chemically classified as an HREE, its adsorption pattern resembled that of LREEs, showing no significant difference from Nd/Ce adsorption in most strains. Moreover, all the strains consistently showed the lowest adsorption capacity for La (groups ε-ζ), indicating limited binding affinity for LREEs. Taken together, these adsorption patterns suggest that these *Bacillus* strains may possess a specific recognition mechanism that preferentially targets HREEs.

In this study, strain DW011 demonstrated significantly higher adsorption rates for HREEs (Tm, Ho, Dy, Tb, Y) than the other tested strains. However, as shown in Section 3.1, DW011 exhibited a comparable adsorption rate for Tb to those of DW012 and PS832, while the other four strains (DW014, DW045, DW051, DW054) showed significantly higher adsorption rates than DW011. This variation may be closely related to differences in surface characteristics among strains and adsorption behaviors under different experimental conditions. Previous studies have shown that REEs are adsorbed onto the cell surface of *Bacillus subtilis* by binding to functional groups [37]. Moreover, the type and quantity of functional groups on the cell wall vary among bacterial species, which can influence their capability to adsorb heavy metals [45]. Furthermore, heavy metal contamination can lead to significant changes in the functional groups on the bacterial surface, which may also contribute to the variations observed in rare earth adsorption among different strains [46]. These differences in functional groups might explain the variations in rare earth adsorption between strain DW011 and the other strains. Secondly, when adsorbing 200 μM Tb(III), the relatively low concentration might have left some unsaturated adsorption sites on bacterial surfaces. In contrast, the higher REE mixture concentration (800 μM, 100 μM for each element) could have led to more complete utilization of adsorption sites, thereby increasing adsorption rates. Additionally, competitive adsorption effects may have played an important role in mixed REE solutions [41]. Competition between different REE ions could influence their adsorption behaviors on bacterial surfaces, enabling DW011 to demonstrate stronger adsorption capacity in mixed REE solutions. Therefore, its superior adsorption performance might result from both full utilization of adsorption sites and competitive adsorption effects. Takahashi et al. found that at lower REE–bacteria ratios, the HREEs adsorbed by cells form complexes with multiple phosphate sites (including phosphoester sites) with larger coordination numbers, while light and medium-weight REEs form complexes with phosphate sites with lower coordination numbers [47]. This provides a better explanation for the preferential adsorption of HREEs by the *Bacillus* strains investigated in the present study. Another plausible explanation for the preferential adsorption of HREEs by the *Bacillus* strains in this study is that the smaller ionic radius of HREEs (Tm(III) 0.94 Å) may facilitate their incorporation into surface micropores of bacterial cells, while larger LREEs like La(III) (1.16 Å) experience reduced binding efficiency due to steric hindrance. Although Y(III) is classified as an HREE, the experimental strains showed similar adsorption capacities for Y(III) as for light rare earth elements (Nd(III), Ce(III), and La(III)). The cause of this phenomenon may lie in the distinct chemical properties of Y(III) compared to typical REEs [32].

Subsequent experiments were conducted to further investigate the potential of strain DW011 for rare earth recovery and to elucidate the adsorption mechanisms of rare earth ions.

### 3.4. Characterization of Strain DW011

#### 3.4.1. Tolerance to Tb(III)

**Figure 4:**
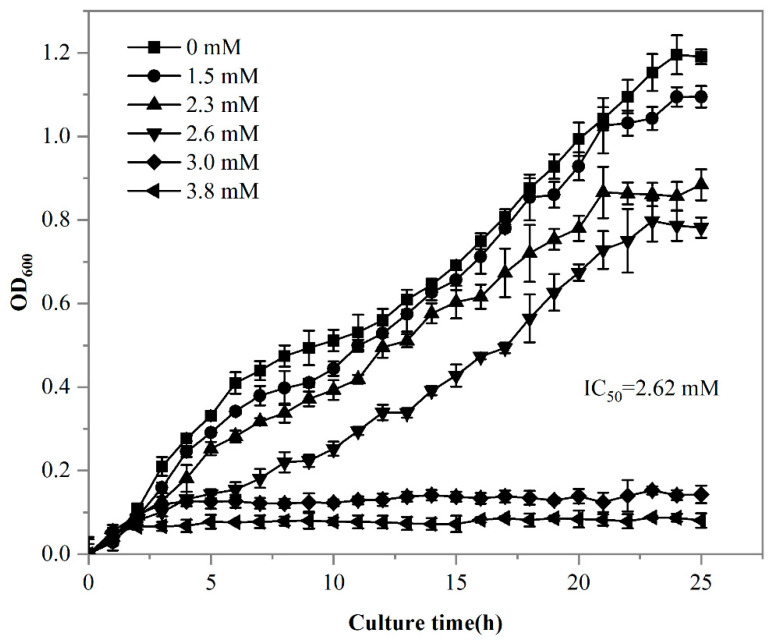
Growth curve of strain DW011 in LB medium with different concentrations of Tb(III). OD_600_ was analyzed in three independent experiments in triplicate, and data are shown as mean ± SD.

It has been demonstrated that living cells exhibit a higher adsorption capacity for REEs compared to dead cells [48]. Given the superior adsorption capacity of living cells, it was essential to evaluate the impact of rare earth stress on strain DW011. Statistical analysis using Tukey’s test demonstrated that extremely significant growth inhibition occurred when Tb(III) concentrations exceeded 1.5 mM (*p* < 0.001), showing a clear dose-dependent pattern. Specifically, the inhibition rates increased progressively from 8.1% at 1.5 mM to 25.6% at 2.3 mM. Furthermore, a complete growth inhibition rate of 71.9% was observed at 3.0 mM, with the maximal inhibition rate reaching 84.9% at 3.8 mM (Figure 4). The 24 h 50% lethal concentration (LC_50_) for DW011 was determined to be 2.62 mM.

It has been demonstrated that REEs inhibit the growth of *Bacillus* at certain concentrations, with higher concentrations intensifying this inhibitory effect [49]. Similarly to strain DW011, other microorganisms also exhibit tolerance to REEs. In a previous study, the thermophilic bacterium *Thermus scotoductus* SA-01 was shown to grow in up to 1.0 mM Eu(III), reaching an OD_600_ of 1.0, but growth ceased when the Eu(III) concentration increased to 2.0 mM [44]. *Streptomyces* W-12 could tolerate 0.2 mM of La, while *Bacillus* W-28 could only tolerate 0.05 mM [50]. Statistical modeling revealed that strain DW011 exhibited significantly higher tolerance to Tb(III) compared to the other strains (*p* < 0.01). The strain maintained measurable growth at 2.62 mM Tb(III) (95% CI: 2.48–2.76 mM), and its tolerance threshold was 2.6–13.1-fold higher than those of *Thermus scotoductus* SA-01 (1.0 mM) and *Streptomyces* W-12 (0.2 mM) (*p* < 0.01). This exceptional tolerance likely stems from its unique cell-surface modification mechanisms, making it particularly valuable for rare earth element biorecovery applications [43]. Furthermore, metal toxicity in bacteria is believed to result from the displacement or substitution of essential surface binding sites and the blocking of critical functional groups on the cell surface [51]. Therefore, the resistance mechanisms of strain DW011 might involve adaptations to mitigate these effects, such as protecting essential binding sites and maintaining functional group integrity. These adaptations could explain its enhanced tolerance to REEs and its potential for rare earth recovery.

#### 3.4.2. Adsorption Kinetics

**Figure 5:**
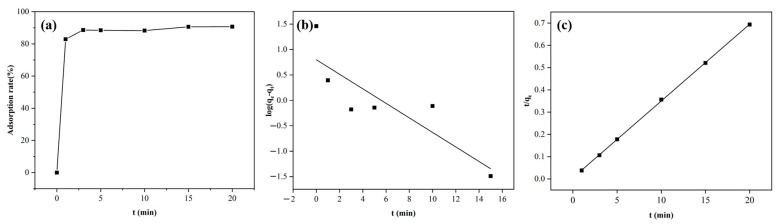
Kinetic models of Tb(III) adsorption by *Bacillus* sp. DW011. The adsorption system consisted of a 250 μL bacterial solution of *Bacillus* sp. DW011 (OD_600_ = 3.0), 50 μL of 2 mM Tb(III) at pH = 7.4, 125 μL of 100mM HEPES, and 75 μL of sterile water. Panel (**a**) depicts the variation in adsorption rate with time, while panel (**b**) presents the fitting results of the pseudo-first-order kinetic model, and panel (**c**) shows the fitting results of the pseudo-second-order kinetic model.

The adsorption capacity of DW011 with different contact times was tested at room temperature. As shown in Figure 5a, the adsorption rate by DW011 increased over time from 0 to 3 min, reaching a plateau at 3 min. Prolonged contact beyond 3 min did not significantly enhance its adsorption capacity.

The increase in the adsorption rate could be attributed to the coordination reaction between the REEs and the coordination groups on the bacterial surface at the initial stage of adsorption, resulting in rapid adsorption to the adsorption sites of the bacteria. As the adsorption time increased, the adsorption sites were gradually occupied and the adsorption rate slowly decreased until it reached equilibrium. Therefore, it was necessary to determine the appropriate adsorption time required to reach equilibrium, which could improve efficiency. The results show that an adsorption time of 3 min was optimal. Notably, this rapid adsorption contrasts with previous reports requiring ≥20 min for equilibrium to be reached [52,53], suggesting the potential of DW011 for scalable applications through reduced retention times and enhanced throughput efficiency, particularly enabling real-time treatment of large-volume, low-concentration REE wastewaters (e.g., mine leachates).

Adsorption kinetics provides information on the rate of adsorption, the time required to reach equilibrium, and the mechanism controlling the process [54]. In order to investigate the adsorption mechanism of strain DW011, the adsorption process was fitted using the pseudo-first-order and pseudo-second-order models. As illustrated in Figure 5b,c, kinetic modeling revealed a superior fit for the pseudo-second-order model (*R*^2^ = 0.9998) compared to the pseudo-first-order model (*R*^2^ = 0.748) according to significant *F*-test results (*F* = 267, *p* < 0.0001). This indicates that the adsorption by DW011 was more consistent with the second-order kinetic model. This agreement with prior studies of *B. licheniformis* and *B. subtilis* reinforces the model’s validity [36,55]. The kinetic parameters obtained from the experimental fitting are presented in Table 2.

The pseudo-second-order kinetic model is mostly used to describe adsorption processes dominated by chemical adsorption, which indicates that the absorption by DW011 may occur through ion exchange or chelation between cells and rare earth ions [20]. In general, pseudo-second-order kinetic models are more suitable for describing the process of biomass adsorption of metal ions than pseudo-first-order kinetic models [41].

#### 3.4.3. SEM-EDS Analysis

**Figure 6:**
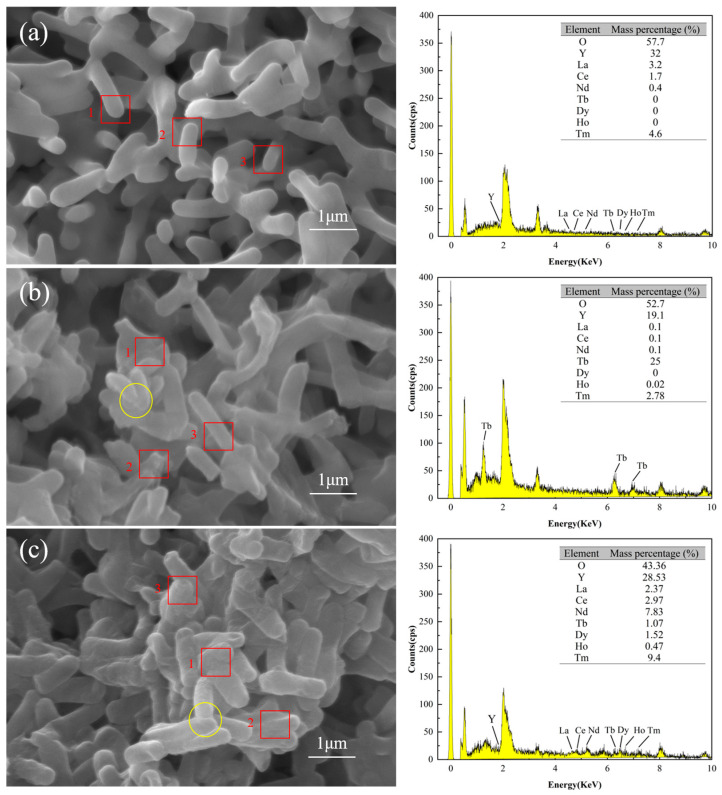
SEM-EDS images of DW011 before and after adsorption. (**a**) DW011 image before adsorption of Tb(III); (**b**) DW011 image after adsorption of Tb(III); (**c**) DW011 image after adsorption of mixed REE solution. Yellow solid-line circles indicate rough cell surface and blurring cell edge after adsorption. EDS data represent arithmetic means of energy intensities from three representative cell surface regions indicated by red rectangles.

SEM/EDS analysis confirmed that the surface of DW011 was complex, with rare earth ions attached. Figure 6a–c illustrate the SEM images and EDS spectra testing of DW011 under three conditions: pre-adsorption, post-adsorption, and post-mixed REE adsorption. Prior to adsorption, the surface of DW011 was observed to be smooth with clear intercellular boundaries. After adsorption, the surface became rough and the cell margins became blurred due to adhesion between cells, indicating the presence of substances adhering to the surface and between cells. A comparison of Figure 6a,b reveals a significant increase in the mass percentage of the Tb element on the surface of DW011 after adsorption, with enhanced characteristic peaks representing Tb, indicating that Tb became newly attached to the surface of DW011. A comparison of Figure 6a,c reveals that the mass percentages of the Tb, Tm, Dy, Nd, Ho, and Ce elements on the surface of DW011 after the adsorption of mixed rare earths increased, and their characteristic peaks were also enhanced. These findings suggest that DW011 adsorbed these elements from the mixed rare earth solution through its cell surface. It is noteworthy that pre-adsorption EDS point analysis indicated an yttrium (Y) content of approximately 32%, but no characteristic yttrium peaks (e.g., Y Lα at ~1.922 keV or Y Kα at ~14.958 keV) were observed in the spectrum. The reported content of 32% was obtained due to calibration drift, which caused the phosphorus (P) Kα peak (2.013 keV) in the low-energy region to be incorrectly attributed to yttrium signals.

The surfaces of microorganisms contain functional groups with metal coordination capabilities, exhibiting high metal adsorption capacities [33]. Similarly, Zhou et al. found surface changes after adsorption of rare earths by *Bacillus* and *Aspergillus niger*, verifying that the rare earths were adsorbed on the cell surface [37]. Liang et al. found that Y was adsorbed by ion exchange on the surface of *Serratia marcescens* [56]. The SEM-EDS analysis indicated that DW011 adsorbed rare earth ions on its surface.

#### 3.4.4. FTIR Analysis

**Figure 7:**
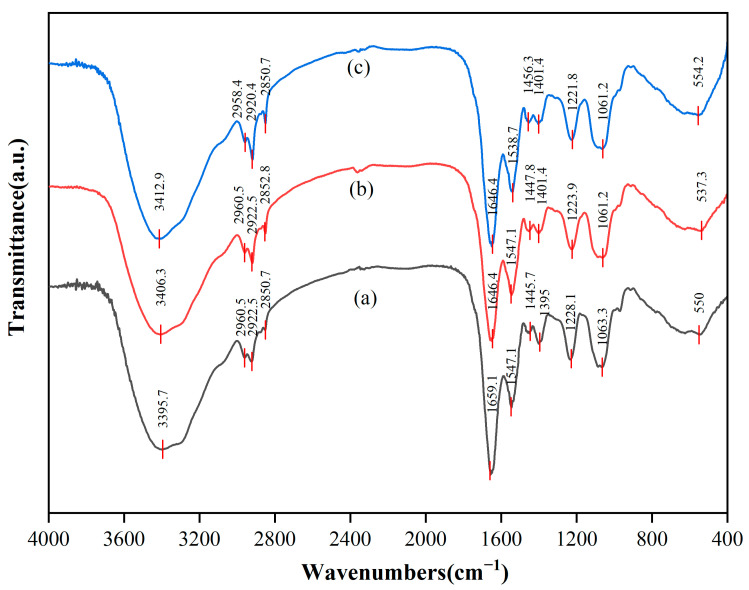
FTIR spectra of DW011 cells. (**a**) Before REE adsorption; (**b**) after Tb(III) adsorption; (**c**) after mixed REE adsorption.

The different functional groups on the surface of cells can be identified as participation in the interaction with REEs by the different positions of absorption peaks in the infrared spectrum. FTIR spectra were recorded before and after the adsorption of biological cells in the range of 4000~400 cm^−1^, as illustrated in Figure 7. The results indicate that the adsorption behavior primarily occurred in the cell wall domain, and different interaction patterns were exhibited for a single rare earth (Tb(III)) and mixed rare earths. For the adsorption of Tb(III), the phosphate groups (P=O) on the cell wall surface played a dominant role. Their stretching vibration peak significantly shifted from 1228.1 cm^−1^ to 1223.9 cm^−1^ (Δν = −4.2 cm^−1^), confirming that Tb(III) bound to the phosphate oxygen atoms in teichoic acid via strong coordination bonds [57]. The carbonyl group (C=O) of peptidoglycan also participated in adsorption, with its stretching vibration peak (amide I band, 1659.1 cm^−1^) red-shifting to 1646.4 cm^−1^ (Δν = −12.7 cm^−1^), indicating that the carbonyl oxygen atoms provide auxiliary coordination [58]. At the same time, the hydroxyl (-OH) peak shifted from 3395.7 cm^−1^ to 3406.3 cm^−1^ (Δν = +10.6 cm^−1^), suggesting that Tb(III) competitively disrupts the existing hydrogen bond network in the cell wall [59]. In contrast, the carboxyl (COO^−^) peak showed a weaker shift (1395 cm^−1^ → 1401.4 cm^−1^, Δν = +6.4 cm^−1^), and the lipid C-H bond peak (2922.5 cm^−1^) remained unchanged (Δν = 0 cm^−1^), indicating that ion exchange and the lipid layer of the cell membrane contribute little to Tb(III) adsorption [60]. Therefore, Tb(III) was mainly localized in the teichoic acid–peptidoglycan complex of the cell wall and did not penetrate into the cell membrane or biological membrane matrix.

Under mixed-rare earth adsorption conditions, it was found that the adsorption followed a competitive stratification mechanism. Heavy rare earths with a high charge density and small ionic radius (e.g., Tm(III)) preferentially occupy and stabilize the phosphate group sites, as indicated by the further red-shift of the P=O peak to 1221.8 cm^−1^ (Δν = −6.3 cm^−1^) [57]. In contrast, light rare earths with larger ionic radii (e.g., La(III), Ce(III), Nd(III), ionic radius &gt; 1.0 Å) were excluded from the phosphate group sites due to spatial steric effects, and instead competed for carboxyl and amino sites in the biomembrane matrix. This resulted in a significant red-shift of the amide II band (N-H) from 1547.1 cm^−1^ to 1538.7 cm^−1^ (Δν = −8.4 cm^−1^), while the carboxyl-related peak shifted from 1445.7 cm^−1^ to 1456.3 cm^−1^ (Δν = +10.6 cm^−1^) [58]. The hydroxyl peak exhibited a further blue-shift to 3412.9 cm^−1^ (Δν = +17.2 cm^−1^), reflecting a more significant disruption of the hydrogen bond network by the synergistic effect of multiple ions [59]. A newly appeared metal–oxygen (M-O) bond peak at 554.2 cm^−1^ (originally near 550 cm^−1^) indicated weaker coordination bonds formed by light rare earths (due to their larger ionic radius, which weakens bond strength). Meanwhile, the lipid C-H bond peak showed only a slight shift (2922.5 → 2920.4 cm^−1^, Δν = −2.1 cm^−1^), further excluding significant hydrophobic embedding in the cell membrane lipid layer [60]. This “ionic radius screening” effect driven by the ionic charge density and radius characteristics of rare earth ions led to a clear stratification of adsorption sites: (i) heavy rare earths with small ionic radii and a high charge density (ionic radius &lt; 0.92 Å) firmly occupied the efficient phosphate group sites on the cell wall surface; (ii) light rare earths with large ionic radii were screened to the lower-efficiency carboxyl/amino secondary sites in the biomembrane matrix (e.g., the adsorption rate of La(III) is only 51.92%); and (iii) the cell membrane lipid layer did not significantly participate in adsorption. The stratification of site efficiency directly explained the phenomenon in the experiment where the adsorption rate of light rare earths was generally 30–40% lower than that of heavy rare earths. In conclusion, the teichoic acid–peptidoglycan network in the cell wall was the core site for the adsorption of rare earths (especially heavy rare earths), with the phosphate groups playing a dominant role. The carboxyl/amino groups in the biomembrane matrix provided secondary adsorption sites for light rare earths, while the cell membrane lipid layer did not significantly participate in the rare earth adsorption process. The differences in adsorption behavior between single and mixed rare earths were primarily driven by a competitive site selection mechanism determined by the characteristics of the rare earth ions.

## 4. Conclusions

In this study, six strains of *Bacillus* with superior adsorption capacity were isolated from ionic rare earth mines. The strains were found to exhibit adsorption selectivity towards REEs, with a pronounced preference for HREEs. Particularly, strain DW011 exhibited the highest selectivity for REEs, with a maximum difference in adsorption rate of 39.53%, providing the first evidence of the adsorption selectivity of *Bacillus* strains toward different REEs. Notably, DW011 exhibited exceptional tolerance, reaching a 24 h IC_50_ value of 2.62 mM. In addition, the adsorption of DW011 conformed to a quasi-secondary kinetic model and reached adsorption equilibrium within 3 min. FTIR spectroscopy revealed a novel site-stratification mechanism of an “ionic radius sieving” effect, that is, HREEs preferentially bound to teichoic acid phosphate groups (P=O shift: −4.2 cm^−1^), while LREEs occupied carboxyl/amino sites (N-H shift: −8.4 cm^−1^). Overall, this study provides a reference point for screening *Bacillus* strains with efficient adsorption and even other HREEs, which could further pave an alternative way for microbial-based application in the recycling of REEs.

## Figures and Tables

**Table 1:** Adsorption rate of REE(III) by *Bacillus* strains.

REE(III)	DW011	DW012	DW014	DW045	DW051	DW054
Tm	91.37 ± 0.74 a α *	51.74 ± 0.16 b α	42.65 ± 0.90 c α	40.54 ± 0.98 c β	54.79 ± 0.47 b β	44.91 ± 0.66 c α
Ho	88.22 ± 0.99 a β	51.13 ± 0.07 b α	38.54 ± 0.11 c β	42.91 ± 0.95 c α	52.63 ± 0.04 b γ	43.20 ± 0.29 c α
Dy	88.84 ± 0.73 a β	52.04 ± 0.15 b α	40.70 ± 0.26 c α	41.65 ± 1.14 c αβ	55.90 ± 0.11 b α	43.49 ± 0.04 c α
Tb	88.99 ± 0.73 a β	49.64 ± 0.27 b β	37.22 ± 0.42 c β	39.32 ± 0.23 c γ	53.06 ± 0.61 b βγ	43.16 ± 1.15 c α
Y	74.25 ± 2.15 a γ	38.01 ± 0.62 b γ	24.48 ± 0.56 d γ	30.38 ± 0.35 c δ	37.88 ± 0.21 b δ	26.15 ± 1.39 d β
Nd	76.87 ± 0.04 a γ	33.75 ± 1.34 b δ	24.12 ± 1.07 c γ	23.81 ± 0.31 c ε	38.50 ± 0.27 b δ	26.42 ± 0.15 c β
Ce	73.13 ± 0.57 a γ	35.05 ± 0.57 b γδ	21.64 ± 0.84 d γ	23.57 ± 0.49 c ε	35.39 ± 0.08 b δ	27.34 ± 0.61 c β
La	51.92 ± 0.07 a δ	24.82 ± 0.67 b ε	17.26 ± 0.21 d δ	14.42 ± 0.21 e ζ	27.52 ± 0.04 c ε	23.12 ± 0.75 d γ

* Data are shown as mean ± SD. Letters a–e in same row and α–ζ in same column indicate significant differences (*p* < 0.05).

**Table 2:** Kinetic modeling parameters for the adsorption by *Bacillus* sp. DW011.

Type	Pseudo-First-Order Kinetic Model			Pseudo-Second-Order Kinetic Model		
q_max_ (mg/mL)	*K*_1_	*q*_e_ (mg/mL)	*R* ^2^	*K*_2_	*q*_e_ (mg/mL)	*R* ^2^
28.832	0.143	2.223	0.748	0.244	28.969	0.9998

## Data Availability

The 16S rRNA sequence data presented in this study are openly available in [NCBI] at [https://www.ncbi.nlm.nih.gov/nuccore/PQ121269.1/], reference number [PQ121269]. Other datasets are available on request due to restrictions.
